# Insulin Regulates Hypoxia-Inducible Factor-1α Transcription by Reactive Oxygen Species Sensitive Activation of Sp1 in 3T3-L1 Preadipocyte

**DOI:** 10.1371/journal.pone.0062128

**Published:** 2013-04-23

**Authors:** Sudipta Biswas, Reshmi Mukherjee, Nisha Tapryal, Amit K. Singh, Chinmay K. Mukhopadhyay

**Affiliations:** Special Centre for Molecular Medicine, Jawaharlal Nehru University, New Delhi, India; University College Dublin, Ireland

## Abstract

Oxygen sensing transcription factor HIF-1 is activated due to accumulation of regulatory subunit HIF-1α by posttranslational stability mechanism during hypoxia or by several other stimuli even in normoxia. HIF-1α is also regulated by NF-kB mediated transcription mechanism. Reactive oxygen species (ROS) act as an important regulator of HIF-1 either by affecting prolyl hydroxylase activity, the critical determinant of HIF-1α stabilization or by activating NF-kB to promote HIF-1α transcription. Insulin is known to activate HIF-1 by a ROS dependent mechanism but the molecular mechanism of HIF-1α regulation is not known so far. Here we show that insulin regulates HIF-1α by a novel transcriptional mechanism by a ROS-sensitive activation of Sp1 in 3T3-L1 preadipocyte. Insulin shows little effect on HIF-1α protein stability, but increases HIF-1α promoter activity. Mutation analyses, electrophoretic mobility shift assay and chromatin immunoprecipitation assay confirm the role of Sp1 in HIF-1α transcription. We further demonstrate that insulin-induced ROS generation initiates signaling pathway involving phosphatidylinositol 3-kinase and protein kinase C for Sp1 mediated HIF-1α transcription. In summary, we reveal that insulin regulates HIF-1α by a novel transcriptional mechanism involving Sp1.

## Introduction

The oxygen sensing transcription factor hypoxia-inducible factor-1 (HIF-1) is a heterodimer of regulatory subunit HIF-1α and constitutive subunit HIF-1β [1]. In oxygen deficiency, HIF-1α expression is regulated by a post-translational protein stability mechanism mediated by a family of prolyl hydroxylases (PHDs) [2], [3]. Upon activation, HIF-1 binds to the hypoxia response elements (HREs) of target genes implicated in energy metabolism, angiogenesis, apoptosis, and iron homeostasis [4]–[6]. In normoxia, HIF-1α is usually unstable due to hydroxylation of two proline residues; Pro^402^ and Pro^564^ that promotes ubiquitination and subsequent proteasomal degradation [7]–[10]. Three different HIF prolyl-hydroxylases termed PHD1, PHD2, and PHD3 are able to hydroxylate HIF-1α using oxygen and 2-oxoglutarate as substrates and iron as well as ascorbate as essential cofactors [2], [3]. Hypoxic conditions lead to HIF-1α stabilization due to inhibition of prolyl hydroxylses and subsequent decrease in HIF-1α ubiquitination and degradation.

HIF-1 is also activated in normoxic condition by several physiological stimuli like growth factors, hormones, cytokines, transition metals and infectious agents [11]–[18]. Insulin regulates several genes important for energy and iron homeostasis mediated by HIF-1 in hepatic and skeletal muscle cells [17], [19]–[22]. Insulin-like growth factor-1 (IGF-1) has been reported to activate HIF-1 by stabilizing HIF-1α protein [23]. Angiotensin II (Ang II) and thrombin also activate HIF-1 in smooth muscle cells [11], [14], [16], [24]. Several transition metals like cobalt, nickel and copper affect PHD activity to activate HIF-1 in various cell types [15], . HIF-1α is also regulated at the transcriptional level mediated by NF-kB [26], [27].

Involvement of reactive oxygen species (ROS) during hypoxia is reported for increased HIF-1α accumulation [28]. However, report of decreased generation of ROS during hypoxia argues against this hypothesis [29]. The report of HIF-1α accumulation by exogenous addition of H_2_O_2_ though supports the role of ROS in HIF-1 activation during normoxic condition. Interestingly, involvement of ROS in HIF-1 activation by several other stimuli like exposures to Ang II [16], [24], [30], thrombin [11], [31] or transition metals [15] has also been reported. In most of these cases ROS was found to affect the PHD activity to stabilize HIF-1α but direct addition of H_2_O_2_ or thrombin induced ROS generation was found to activate NF-kB for HIF-1α transcription [11], [26], [31]. HIF-1 activation is also dependent on hydroxylation of HIF-1α asparagine 803 residue by factor inhibiting HIF (FIH) that controls C-terminal transactivation domain activity [32]. Surprisingly, HIF asparaginyl hydroxylation was found to be more sensitive to low concentrations of H_2_O_2_ than prolyl hydroxylation [33].

HIF-1 activation is associated with the obese adipocytes in which insulin plays a major role [34]. A recent report shows increased HIF-1α mRNA level in adipose tissue in response to insulin [34] but the molecular mechanism of this regulation is not understood. Our earlier report established the role NADPH oxidase mediated ROS generation in insulin-induced activation of HIF-1 [19] but the precise role of ROS in this regulation remained unclear. Interestingly, an essential role of ROS generation in insulin-induced gene expression in adipocytes was established earlier [35]. These findings led us to investigate the molecular mechanism of insulin induced HIF-1α mRNA level and the role of ROS therein in adipocytic 3T3-L1 cells. Here we show that insulin-induced NADPH-oxidase-generated ROS is involved in phosphatidylinositol 3-kinase (PI3K) and protein kinase C (PKC) dependent Sp1 activation that binds a GC-rich region in HIF-1α promoter. This study not only reveals that unlike hypoxia or most of other stimuli the insulin-induced HIF-1α expression is regulated primarily by a novel transcriptional mechanism but also demonstrates a differential role of ROS in HIF-1 activation other than affecting PHD activity or NF-kB activation.

## Results

### Insulin Induces HIF-1α Accumulation in 3T3-L1 Preadipocyte

**Figure 1:**
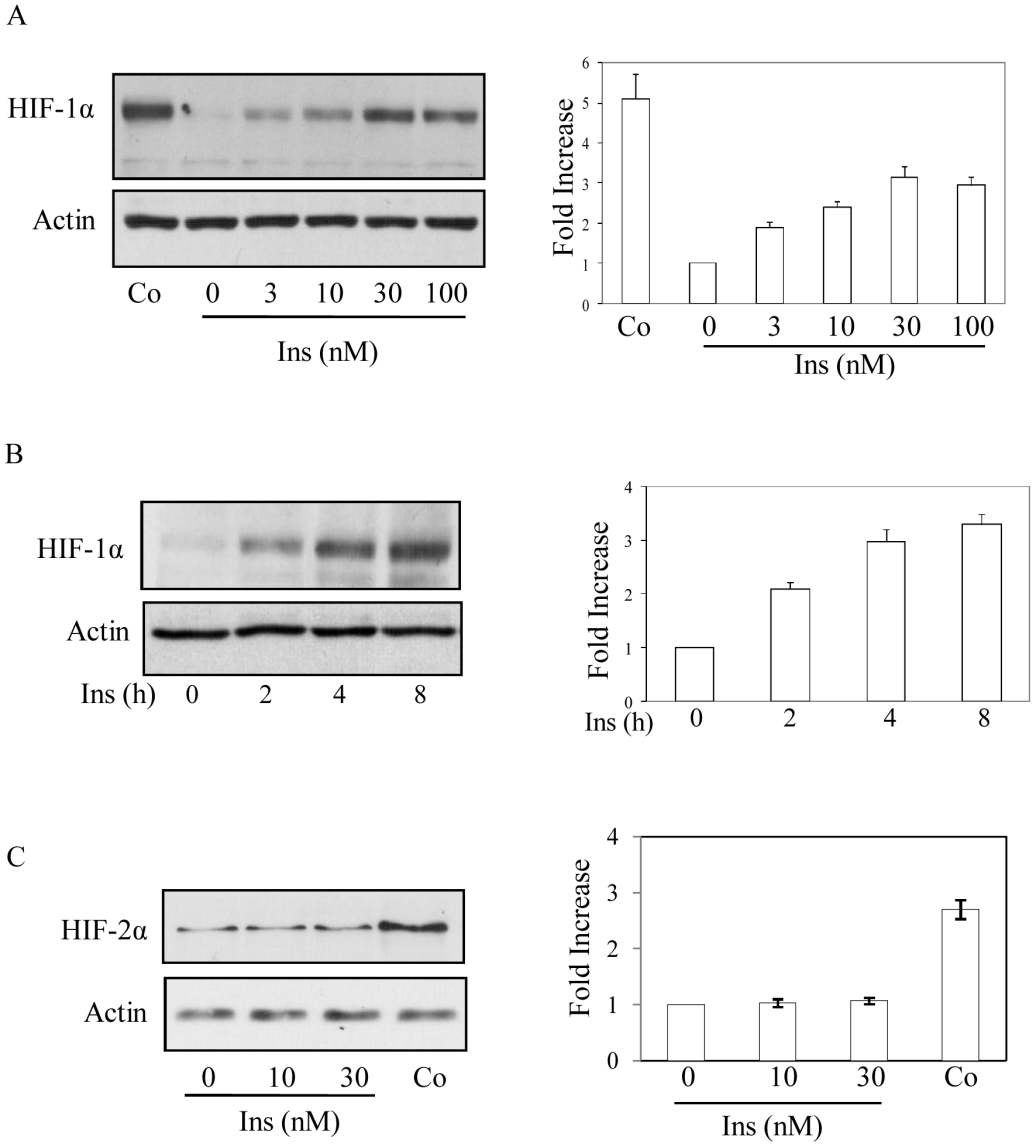
Insulin regulates HIF-1α accumulation. (A) Serum deprived 3T3-L1 cells were either treated with insulin (0–100 nM) or cobalt chloride (150 µM) for 8 h. Immunoblot analysis was performed with HIF-1α (left upper panel) and actin (left lower panel) antibody using nuclear extracts. Right panel shows densitometric analysis of three independent experiments. (B) Similarly, immunoblot analysis was performed using HIF-1α (left upper panel) and actin (left lower panel) antibody in nuclear extracts isolated from insulin (30 nM, 0–8 h) treated cells. Right panel shows densitometric analysis of three independent experiments. (C) Serum deprived 3T3-L1 cells were either treated with insulin (0–30 nM) or cobalt chloride (150 µM) for 8 h. Immunoblot analysis was performed with HIF-2α (left upper panel) and actin (left lower panel) antibody using nuclear extracts. Right panel shows densitometric analysis of three independent experiments.

**Figure 2:**
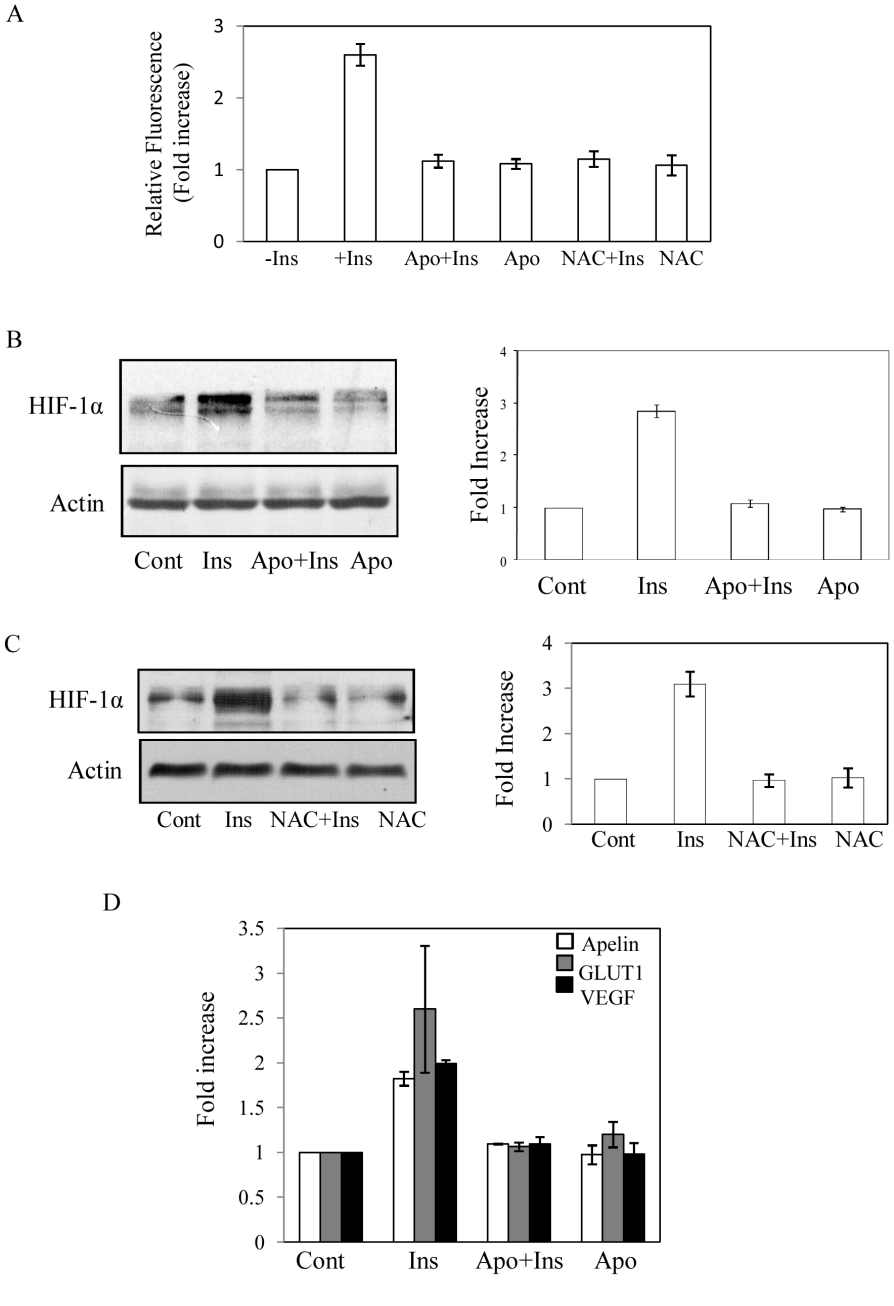
Role of ROS in Insulin-induced HIF-1α accumulation. (A) To verify ROS generation by insulin, cells were serum deprived and followed by 30 min apocynin (300 µM) or NAC (5 mM) treatment. Then incubated with 2′, 7′-DCF-DA (5 µM) for 30 min at 37°C in the dark prior to the insulin treatment (30 nM). After 10 min cells were washed twice with 1×PBS and fluorescence was measured. Results are SD of four independent experiments performed as triplicate. (B) Cells were treated with apocynin (300 µM) for 30 min before addition of insulin (30 nM) for 6 h and Western blot analysis was performed using HIF-1α antibody and actin antibody (left panels). In right panel results are shown as SD from densitometric analysis of three independent experiments. (C). Similarly, cells were treated with NAC (5 mM) 30 min, then treated with insulin (30 nM) for 8 h and Western blot analysis was performed using HIF-1α and actin antibodies (left panels). Right panel shows SD of densitometric analysis of three independent experiments. (D). Cells were serum deprived and treated with insulin (30 nM). Apocynin (300 µM) was added 30 min prior to addition of insulin. After 8 h of insulin treatment total RNA was isolated and quantitative reverse-transcriptase PCR was performed using specific primers of apelin, GLUT1 and VEGF. Results are representative of SD of four independent experiments.

To determine the effect of insulin on HIF-1α accumulation, serum-deprived cells were treated with insulin (0–100 nM) and HIF-1α levels were determined in nuclear extracts by immunoblot analysis. A dose dependent increase in HIF-1α (about 3-fold) was detected up to 30 nM of insulin treatment (Fig. 1A). Further increase in insulin concentration showed no additional increase in HIF-1α accumulation. Cobalt chloride (150 µM) was used as a positive control that increased HIF-1α accumulation about 5-fold (Fig. 1A). HIF-1α accumulation was observed as early as 2 h and maximum increase was detected at 4 h that remained up-regulated at least up to 8 h (Fig. 1B). To find that whether insulin had any role in HIF-2α expression, nuclear extracts isolated from insulin treated cells (0–30 nM) were subjected to Western blot analysis. While cobalt chloride treated cells showed about 3-fold increase in HIF-2α protein level but no significant difference was observed by insulin treatment (Fig. 1C). To determine whether NADPH oxidase (NOX)-mediated ROS generation was involved in HIF-1α accumulation in adipocytic cell, specific NOX inhibitor apocynin (Apo) was used. Initially, insulin-induced ROS generation was determined. Results showed a strong increase in insulin-induced ROS generation that was blocked by Apo (Fig, 2A). In a similar condition, insulin-induced HIF-1α expression was blocked by Apo treatment (Fig. 2B). A similar result was also obtained with another NOX-mediated ROS blocker diphenyleneiodonium chloride (DPI) (data not shown). Moreover, pretreatment of a general antioxidant N-acetyl cysteine (NAC) blocked both the insulin-induced ROS generation and HIF-1α accumulation (Fig. 2A & 2C). We also found that HIF-1 target genes like apelin, Glut1 and VEGF expressions were increased by insulin. Apo pretreatment blocked insulin-induced expressions of these target genes as detected by quantitative reverse-transcriptase PCR (Fig. 2D). These results suggest that insulin induces HIF-1α accumulation in a ROS-sensitive mechanism in 3T3-L1 adipocytic cells.

### Insulin does not Promote HIF-1α Protein Stability

**Figure 3:**
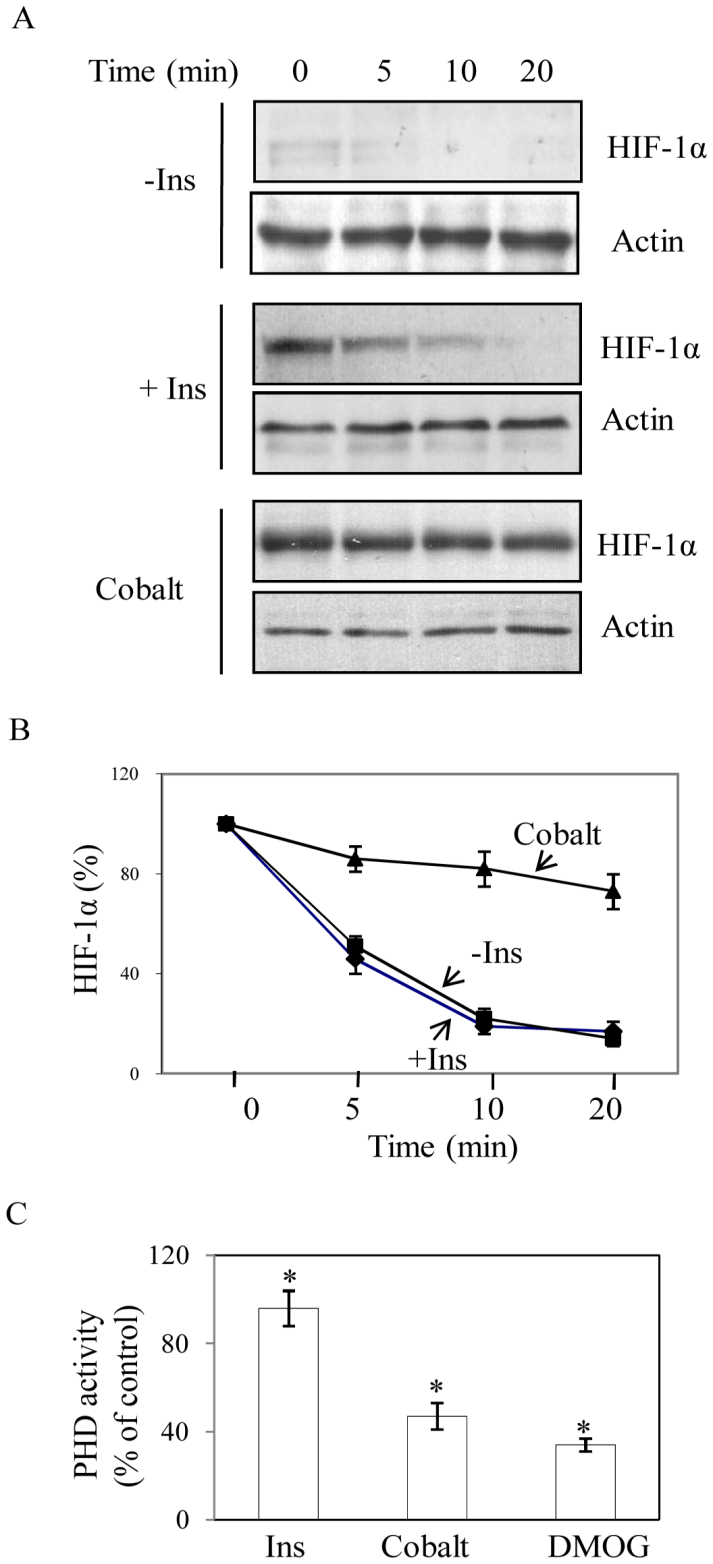
Role of insulin on HIF-1α stabilization. (A) Cells were treated for 6 h with only media (-Ins), 30 nM insulin or cobalt chloride (150 µM). Then cycloheximide (10 µg/ml) was added to stop new protein synthesis. Nuclear extracts were isolated after 0, 5, 10 and 20 min of cycloheximide treatment and immunoblot analyses were performed with HIF-1α and actin antibody. (B) Quantitative representation of HIF-1α degradation (normalized with actin) as mentioned in above experiment. Result represents densitometric analysis of three independent experiments. (C) Prolyl hydroxylase assay was performed as a measure of 2-oxoglutarate (2-OG) consumption in cytoplasmic extracts isolated from control, insulin (30 nM), cobalt chloride (150 µM) and DMOG (0.5 mM) treatment for 4 h. Results are expressed as SD from three independent experiments, *p<0.04, ANOVA.

To find the mechanism of HIF-1α accumulation we determined the rate of HIF-1α protein degradation in absence or presence of insulin as described earlier [18]. Initially, cells were treated with insulin for 6 h and then cycloheximide was added to stop protein synthesis. After 0, 5, 10 and 20 minutes of cycloheximide addition nuclear extracts were isolated and Western blots were performed with HIF-1α and actin antibodies. Well known hypoxia mimetic Cobalt chloride was used as a positive control. Results showed a strong increase in HIF-1α stability by cobalt chloride (Fig. 3A) but insulin did not show any significant change in HIF-1α stability compared to untreated cells (Fig. 3A–B). Since, cellular PHD activity inversely affects HIF-1α stabilization [8]–[9] and intracellular ascorbate is an important cofactor for PHD [15], [30], we determined cellular PHD activity and ascorbate concentration [30] after insulin treatment. Results showed no significant change in PHD activity by insulin compared to untreated cells whereas cobalt chloride and DMOG blocked PHD activity by about 60% and 65% respectively (Fig. 3C). Intracellular ascorbate concentration was unaltered by insulin treatment, whereas CoCl_2_ treatment depleted ascorbate level about 55% (data not shown). These results suggest that HIF-1α stabilization mechanism does not significantly contribute in insulin-induced HIF-1α accumulation.

### Insulin Promotes HIF-1α Transcription

**Figure 4:**
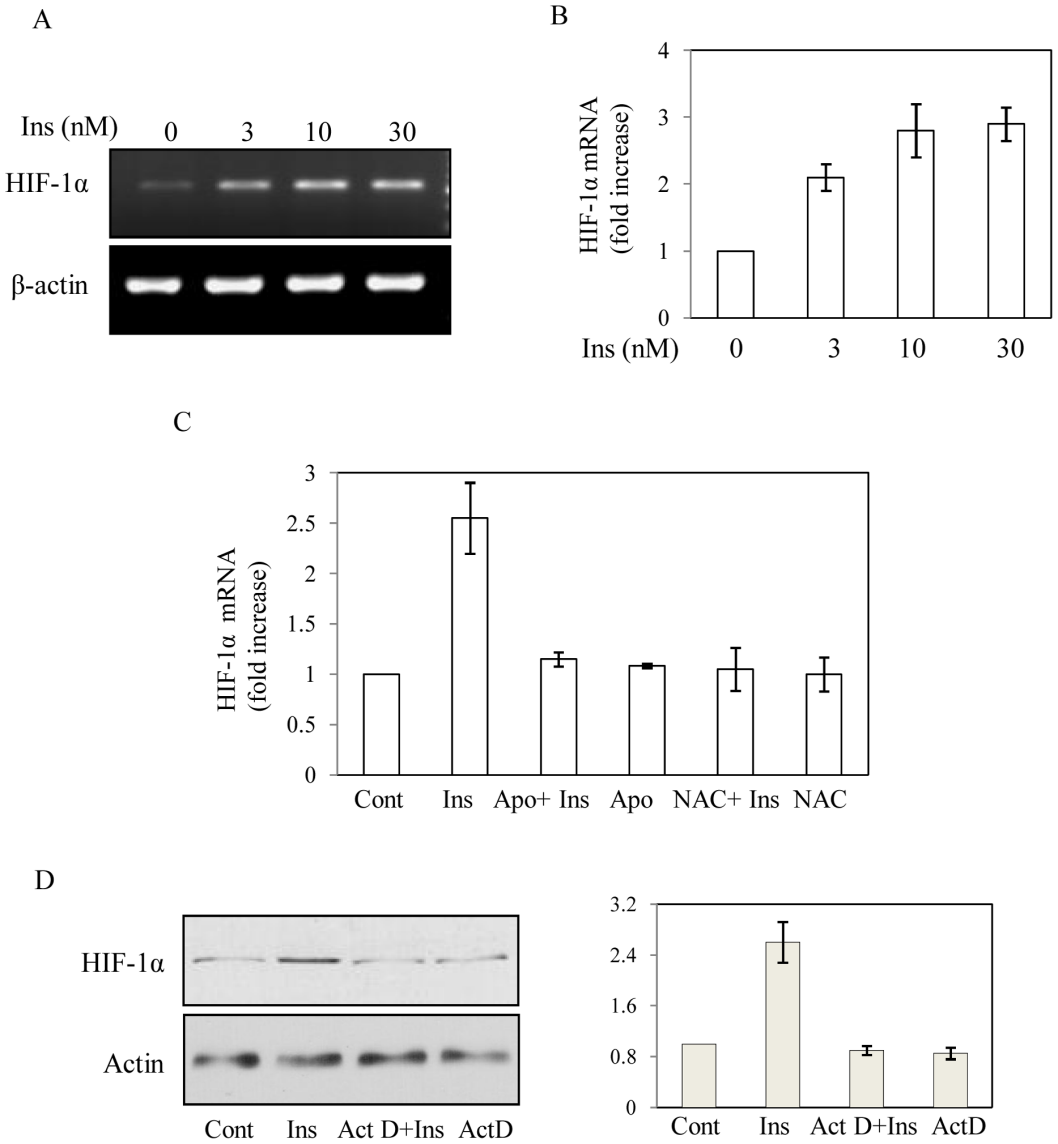
Effect of insulin on HIF-1α mRNA expression. (A) Total RNA was isolated from cells after 4 h of insulin (0–30 nM) treatment and semi-quantitative RT-PCR was performed using mouse specific HIF-1α and β-actin primers. All the PCRs (24 cycles) were performed within the linear range of gene expressions standardized separately. Data is representative of one of the three independent experiments (B) Real time RT-PCR was performed from total RNA isolated from cells after 4 h of insulin (0–30 nM) treatment using mouse specific HIF-1α and β-actin primers obtained from Applied Biosystems. Results are expressed as SD from three independent experiments. (C) Similarly, Real time RT-PCR was performed using mouse specific HIF-1α and β-actin primers from total RNA isolated from cells after 4 h of insulin (0–30 nM) treatment. In some cases Apocynin (300 µM) or NAC (5 mM) was added 30 min prior to insulin treatment. Results are expressed as SD from three independent experiments. (D) Cells were treated with insulin (30 nM, 8 h). In some cases Actinomycin D (5 µg) was added 30 min prior to insulin treatment and Western blot analyses were performed for HIF-1α and actin in nuclear extracts (left panels). Results are expressed as SD from three independent experiments after densitometric analysis (right panel).

**Figure 5:**
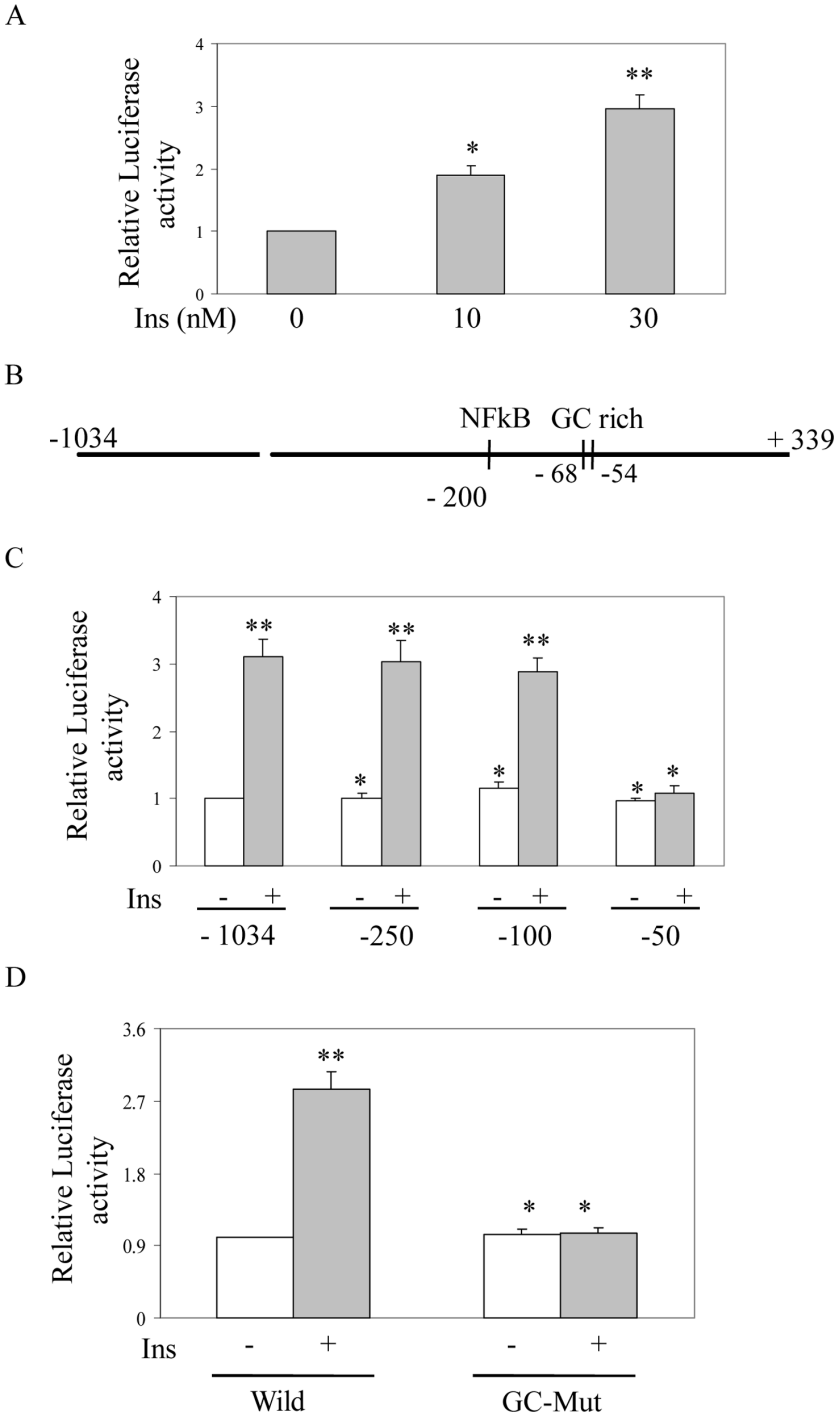
Determination of insulin-responsive element by deletion and mutation analyses of HIF-1α gene 5′-flanking region. (A) Insulin regulates HIF-1α promoter activity. Mouse HIF-1α promoter containing luciferase plasmid was transfected into subconfluent 3T3-L1 cells along with SV40 promoter-linked β-galactosidase. After insulin (0–30 nM) treatment for 8 h, luciferase assay was performed in cell lysates and normalized with β-galactosidase activity. Results are expressed as SD of four independent experiments performed in triplicate, *p<0.05; **p<0.07, ANOVA. (B) A linear map of the promoter region of mouse HIF-1α showing consensus transcription factor binding sites. (C) Mapping of insulin-responsive element in HIF-1α promoter by deletion analysis. Chimeric pGL3-basic vectors were constructed to contain the proximal 1034, 250, 100 or 50 bp of the 5′-flanking region of HIF-1α (upstream of the transcription initiation site) driving luciferase gene. All constructs were transfected along with a plasmid containing β-galactosidase to monitor transfection efficiency. Transfected cells were treated with insulin (30 nM) for 8 h, or were left untreated. Luciferase activity in cell extracts was measured and normalized for β-galactosidase activity. Results are expressed as SD of four independent experiments performed in triplicate, *p<0.04, **p<0.07, ANOVA. (D) Identification of insulin responsive element by site-directed mutation analysis. The 250 bp upsteam segment of the HIF-1α 5′-flanking region containing the putative Sp1 binding GC-rich region was ligated upstream of the luciferase gene in pGL3-basic vector (Wild). Similarly, a second construct containing the same segment but with the core of the putative Sp1 binding GC-rich region mutated (GC-Mut) was made. These constructs were transfected (along with a β-galactosidase plasmid) and were treated for 8 h with insulin (30 nM), or were left untreated. Luciferase activity in cell extracts was assayed and normalized with β-galactosidase activity. Results are expressed as SD of three independent experiments performed in triplicate, *p<0.03; **p<0.07, ANOVA.

To further determine the mechanism of insulin induced HIF-1α accumulation; we examined HIF-1α transcript level by reverse transcriptase PCR. With increase in insulin concentration (0–30 nM) HIF-1α transcript levels were increased (Fig. 4A) to maximum about 3-fold as detected by qPCR analysis (Fig. 4B). When cells were incubated with NOX inhibitor Apo prior to insulin treatment, insulin-induced HIF-1α transcript level was blocked as detected by qPCR analysis (Fig. 4C). A similar data was also obtained by another NOX-inhibitor DPI (Figure S1) and by pretreatment with general antioxidant NAC (Fig. 4C). These data suggest that insulin-induced NOX-mediated ROS generation is involved in insulin-induced increase in HIF-1α transcript level. Our earlier data suggest that insulin has little role in regulating PHD activity (Fig. 3) indicating that HIF-1α transcription is primarily responsible for its increased expression. To further confirm that global transcription inhibitor Actinomycin D (Act D) was added prior to insulin treatment and the effect was verified by Western blot analysis. Result showed a complete blocking of insulin-induced HIF-1α protein accumulation by Act D (Fig. 4D). Then to determine the role of insulin on HIF-1α transcription we cloned its promoter in pGL3-basic vector at the upstream of luciferase gene, transfected into cells and performed luciferase assay after insulin treatment. Simultaneously, β-galactosidase under the control of SV40 promoter [19] was cotransfected to monitor transfection efficiency. A dose dependent increase in luciferase activity confirmed the involvement of transcriptional mechanism in insulin induced HIF-1α accumulation (Fig. 5A). To understand the molecular mechanism, progressive deletion mutants from 5′-end of HIF-1α promoter region were constructed in pGL3-basic vector, transfected into cells and treated with insulin. ROS-sensitive NF-kB mediated transcriptional activation of HIF-1α was reported earlier [26] but deletion of NF-kB binding site (Fig. 5B) did not affect the increased insulin-induced promoter activity (Fig. 5C). The chimera containing −100 nucleotides (from transcription start site) also showed insulin-induced luciferase activity, but deletion of nucleotides from −100 to −50 containing a GC-rich region completely blocked insulin-induced luciferase activity (Fig. 5C). These results indicate that Sp1 binding GC-rich region [−68 to −54] may participate in insulin-induced HIF-1α transcription. Mutation of the same GC-rich region (GC-Mut) blocked insulin induced luciferase activity (Fig. 5D) confirming involvement of GC-rich putative Sp1 binding site in HIF-1α transcription by insulin.

### Identification of Sp1 for Insulin-induced HIF-1α Transcription

**Figure 6:**
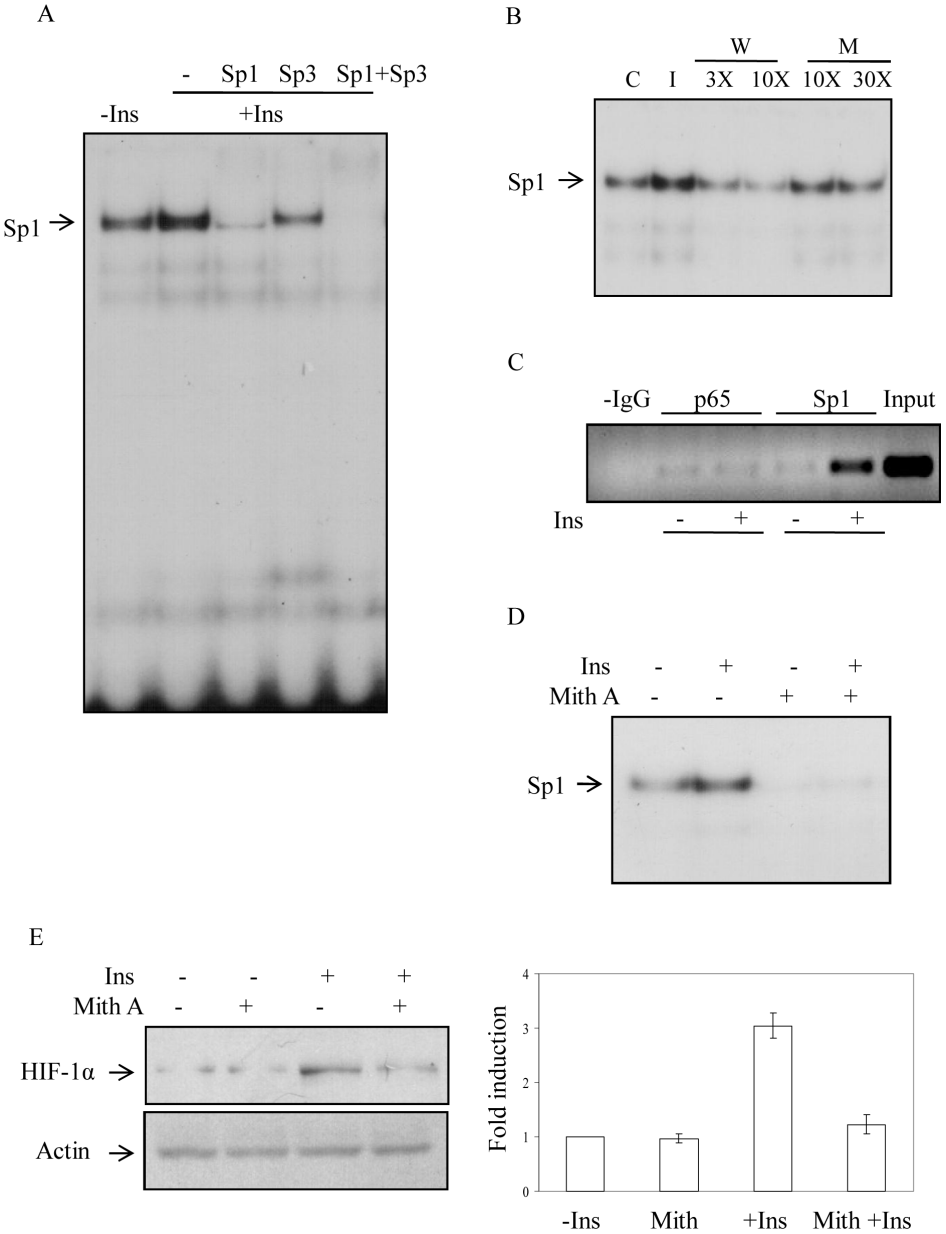
Identification of Sp1 as insulin-stimulated transcription factor to bind HIF-1α promoter region. (A) EMSA was performed to determine HIF-1α promoter binding complexes. 3T3-L1 cells were treated for 4 h with insulin (30 nM). Nuclear extracts were mixed with a ^32^P-labeled, double-stranded 24-mer probe containing putative Sp1 binding GC-rich region of HIF-1α promoter. Similarly, nuclear extracts were incubated with ^32^P-labeled, 24-mer probe in the presence of 2 µl of anti-Sp1, anti-Sp3, or both for 30 min. Probe-bound complexes were resolved by 5% nondenaturing PAGE and visualized by autoradiography. The position of the DNA-protein complex is indicated by arrow. (B) To determine specificity of the binding radiolabeled probe was pre-mixed with unlabeled, annealed, 24-mer oligonucleotide competitor at 3- or 10-fold molar excess before addition to the nuclear extracts for wild-type (W). For mutant (M) 10- or 30-fold molar excess probes were used. After the incubation, mixtures were subjected to 5% nondenaturing PAGE and autoradiography. The position of the Sp1/Sp3 -DNA complex is indicated by arrow. (C) Nuclear extracts prepared from insulin (30 nM) treated and untreated cells were subjected to chromatin immunoprecipitation assays in which anti- p65 and anti-Sp1 antibodies were used. PCR products were analyzed on 1.5% agarose gel with ethidium bromide staining. (D) Cells were treated with mithramycin A (Mith A, 0.5 µM) for 30 min before insulin (30 nM) treatment for 4 h. Nuclear extracts were isolated and EMSA was performed using ^32^P-labeled, double-stranded 24-mer probe containing Sp1 binding GC-rich region of HIF-1α promoter. All these results (A–D) are representative of one of three independent experiments with similar result. (E) Immunoblot analyses were performed in nuclear extracts as described in previous experiment (D) using HIF-1α (left upper panel) and actin (left lower panel) antibodies. Right panel shows SD of densitometric analysis of three independent experiments.

To confirm the involvement of Sp1 we performed EMSA using radiolabeled 24 nucleotide double stranded probe containing GC-rich region of HIF-1α promoter. A strong induction of DNA-binding complex was detected in nuclear extract isolated from insulin-induced cells (Fig. 6A, lane 2) that was supershifted in presence of Sp1 antibody (lane 3). Sp1 often forms a complex with Sp3 to bind GC-region of the DNA [36]. In presence of Sp3 antibody a significant reduction of DNA-binding complex was observed (lane 4) suggesting Sp3 might be the part of the complex. Accordingly, in presence of both Sp1 and Sp3 antibodies the DNA-binding complex was completely abolished with appearance of supershifted band (lane 5) further supporting that both Sp1 and Sp3 were part of the DNA binding complex. Interestingly, slower mobility band was not very prominent even though insulin-induced DNA binding complex was completely disappeared in the presence of Sp1 and Sp3 antibodies implying that the part of the complex might not entered into the gel. The specificity of the binding was verified by addition of 3× and 10× molar excess of unlabeled wild type probe (W) that blocked complex formation with increase in concentration of the probe (Fig. 6B, lanes 2–3) but unlabeled probe containing mutation at Sp1-binding GC nucleotides (M) showed only a marginal effect even in 30× molar excess (Fig. 6B, lanes 4–5). We further confirmed the involvement of Sp1 in vivo by ChIP analysis (Fig. 6C). ChIP analysis using antibody of NF-kB component p65 showed no effect with insulin treatment (Fig. 6C). To further verify the involvement of Sp1, mithramycin A, a known inhibitor of Sp1 binding to GC-rich region [37] was used. We initially verified the ability of mithramycin A in blocking of Sp1 binding to the GC-rich region of HIF-1α promoter by EMSA. More than 95% binding of Sp1 was blocked by mithramycin A (0.5 µM) in both insulin treated and untreated condition (Fig. 6D). Pretreatment of similar concentration of mithramycin A for 30 min blocked more than 90% of insulin-induced HIF-1α accumulation detected by Western blot analysis confirming the role of Sp1 in insulin-induced HIF-1α transcription (Fig. 6E).

### Insulin-induced ROS Generation is Involved in PI3K-PKC Mediated Activation of Sp1

**Figure 7:**
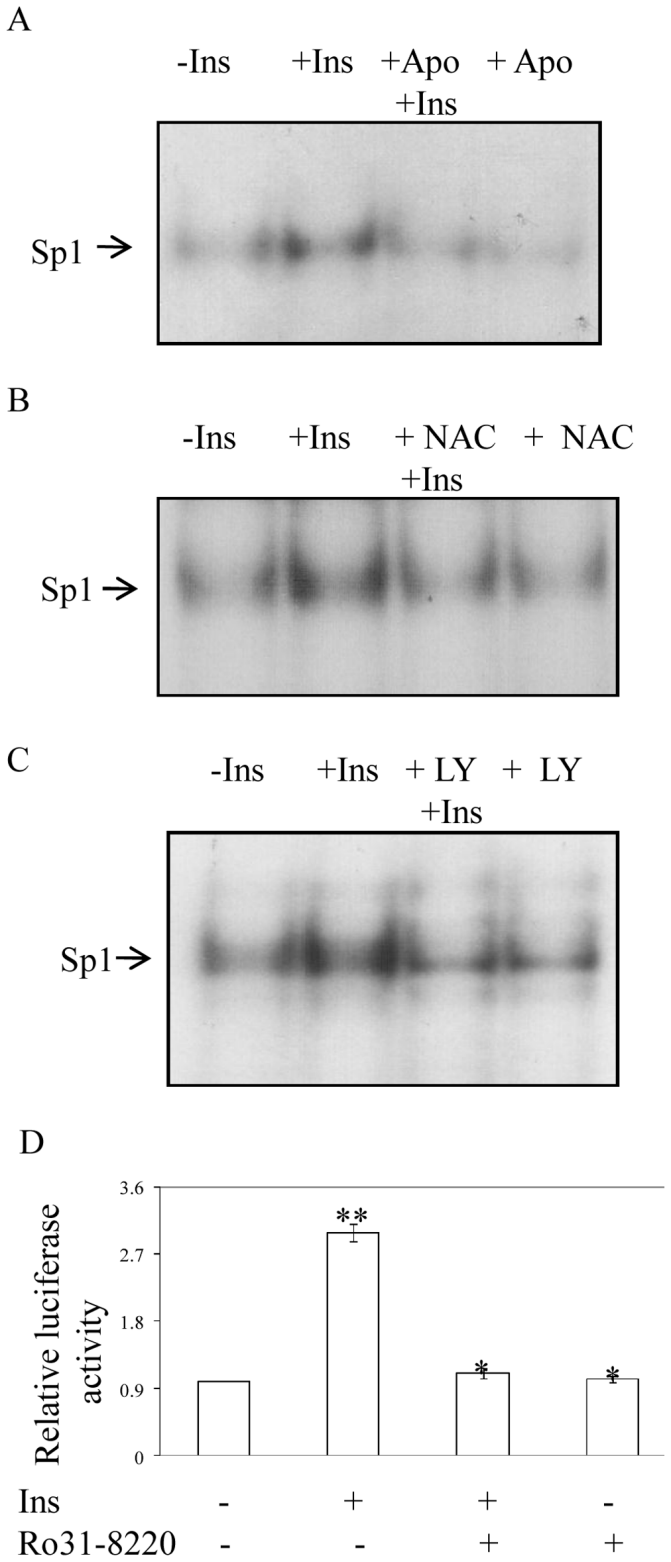
Involvement of ROS, PI3K and PKC in insulin-induced Sp1 activation. Role of ROS in Sp1 binding to HIF-1α promoter. Cells were pretreated with Apo (300 µM) (A) or NAC (5 mM) (B) for 30 min prior to insulin (30 nM) treatment for 4 h. Nuclear extracts were isolated and EMSAs were carried out using ^32^P-labeled, double-stranded 24-mer probe containing Sp1 binding site of HIF-1α promoter. Results are representative of one of the three independent experiments with similar results. (C) Similarly, EMSA was carried out using nuclear extract isolated from cells treated with LY294002 (20 µM, 30 min) prior to insulin treatment (30 nM for 4 h). (D) The 250 bp upsteam segment of the HIF-1α 5′-flanking region containing the Sp1 binding GC-rich region was ligated upstream of the luciferase gene in pGL3-basic vector and was transfected along with a β-galactosidase plasmid. Cells were pretreated for 30 min with Ro31-8220 (5 µM) prior to insulin treatment (30 nM, 8 h). Luciferase assay was performed in cell lysate to test HIF-1α promoter activity and normalized with β-galactosidase activity. Results are expressed as SD of three independent experiments performed in triplicate, *p<0.03; **p<0.04, ANOVA.

Our earlier data (Fig. 2 and 4C) confirmed the involvement of ROS generation in insulin-induced HIF-1α synthesis. To find the precise role of ROS on insulin induced HIF-1α transcription, we blocked ROS generation by Apo and examined its effect on binding of Sp1 to HIF-1α promoter region by EMSA. The result showed complete blocking of insulin-induced Sp1 binding to the HIF-1α promoter by Apo treatment (Fig. 7A). A similar result was also obtained in presence of another antioxidant NAC (Fig. 7B). Since, involvement of phosphatidyl 3-kinase (PI3K) was reported for insulin-induced HIF-1 activation [19], [21], we verified the involvement of PI3K on Sp1 activation. When cells were pretreated with specific PI3-kinase inhibitor LY294002 (20 µM), insulin-induced Sp1-DNA binding was also blocked (Fig. 7C). There are reports of involvement of protein kinase C on insulin-induced Sp1 activation [38]; so, to find the downstream kinase involved after PI3K, cells were pretreated with a pan-specific PKC inhibitor Ro31-8220 and HIF-1α promoter activity was examined. The result showed that insulin-induced HIF-1α promoter activity was blocked by PKC inhibitor R031-8220 (Fig. 7C). These results demonstrate that insulin-induced ROS generation activates PI3K- PKC pathway for Sp1 activation.

**Figure 8:**
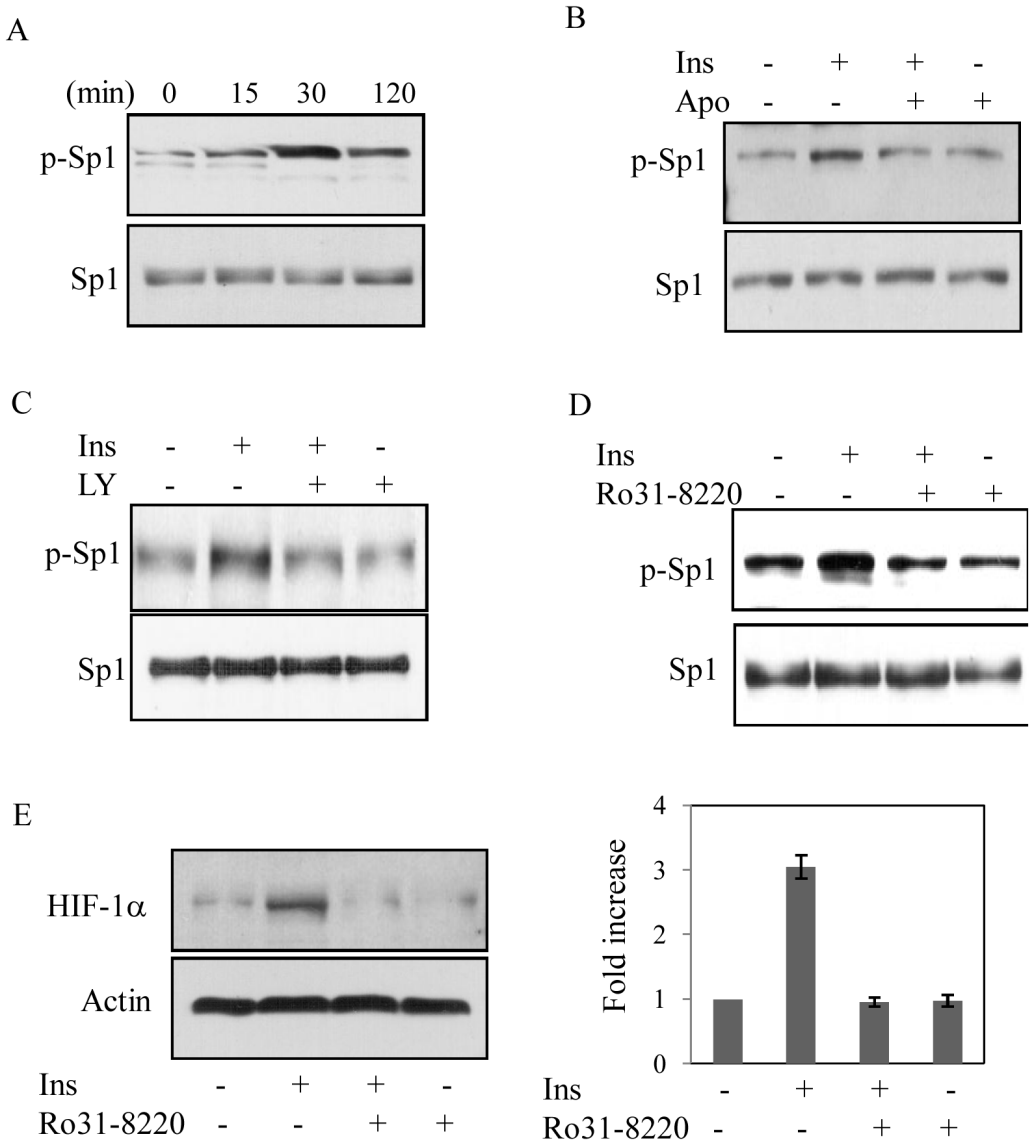
Role of Sp1 phosphorylation in insulin-induced HIF-1α synthesis. (A) Cells were treated with insulin for a different period of time, cell lysates were prepared and subjected to immunoblot analysis using phospho-Thr specific Sp1 antibody (upper panel) or Sp1 antibody (lower panel). (B). Cells were treated with Apo (300 µM) for 30 min prior to insulin treatment (30 nM) for 30 min. Immunoblot analyses with either phospho-Sp1 antibody (upper panel) or Sp1 antibody was performed using cell lysate. Similarly, cells were pretreated with LY294002 (20 µM) (C) or Ro31-8220 (5 µM) (D) for 30 min and then treated with insulin for 30 min and immunoblot analyses were performed using phospho-Sp1 antibody (upper panels) or Sp1 antibody (lower panels). All these experiments (A-D) were performed at least three times with similar results obtained. (E) Immunoblot analysis was performed in nuclear extracts isolated from cells pretreated with Ro31-8220 (5 µM) for 30 min and then subjected to insulin treatment (30 nM, 8 h). HIF-1α (left upper panel) and actin (left lower panel) expressions were detected as described earlier. Right panel shows SD of densitometric analysis of three independent experiments.

It is now well established that Sp1 activation often depends on its phosphorylation [36]. To determine the role of insulin on Sp1 phosphorylation in 3T3-L1 cells, Western blot analysis was performed with phospho-Sp1 specific antibody that could detect phosphorylation of a conserved Thr residue of Sp1. A time dependent increase in Sp1 phosphorylation was detected maximally at 30 min (Fig. 8A). Since, PKC is a Ser-Thr kinase [36], we hypothesized that PKC is involved in Sp1 phosphorylation. In that case, inhibitors of ROS, PI3K or PKC should block insulin induced Sp1 phosphorylation. When we pretreated cells with either Apo or LY294002 or Ro31-8220, insulin-induced Sp1 phosphorylation was blocked (Fig. 8B-D). Finally, blocking of PKC by Ro31-8220, which blocked HIF-1α promoter activity (Fig. 7D) and Sp1 phosphorylation (Fig. 8D) also blocked insulin induced HIF-1α synthesis (Fig. 8E). All these results strongly suggest the involvement of ROS-induced Sp1 activation mediated by PI3K-PKC pathway for insulin-induced HIF-1α transcription.

## Discussion

Stabilization of HIF-1α is the key molecular event for HIF-1 activation in response to hypoxia [1] or several other stimuli like angiotensin II [16], [24], [30], IGF-1 [23] and transition metals [15], [25]; whereas, H_2_O_2_ or lipopolysaccharide (LPS) treatment promotes NF-kB mediated HIF-1α transcription for HIF-1 activation [26], [39]. Although, insulin was found to activate HIF-1 [17], [19]–[21], the key regulatory mechanism of the activation was not established so far. In this study we reveal that insulin regulates HIF-1α by a novel transcriptional mechanism involving Sp1 in 3T3-L1 preadipocytes. Earlier, we reported that NADPH-oxidase mediated ROS generation and subsequent PI3K activation was needed for HIF-1 activation [19]. A recent study confirmed the involvement of NOX4 mediated ROS generation in insulin-induced HIF-1 activation in human microvascular endothelial cell [40]. Our current study reveals that ROS generation is important for PI3K and PKC mediated Sp1 activation for binding to the GC-rich region in HIF-1α promoter in response to insulin. This study establishes a novel mechanism of transcriptional regulation of HIF-1α that depends on insulin-induced ROS generation by NADPH oxidase.

During hypoxia, ROS generation from mitochondrial complex III was reported to be involved for inhibiting cellular PHD activity to stabilize HIF-1α [28]. However, recent finding of decreased generation of ROS during hypoxia raised concerns regarding direct involvement of ROS in affecting PHD activity [29]. There are reports of HIF-1α stabilization by inhibition of PHD activity due to mitochondrial ROS generation by Ang II in vascular smooth muscle cell [24], [30]. Transition metals like cobalt and nickel also affect PHD activity by depleting cellular ascorbate level [15]. So, ROS-mediated inhibition of PHD activity was reported as major cellular mechanism in both hypoxic and normoxic conditions. In contrast, we detected that insulin increased HIF-1α transcription by a ROS sensitive Sp1 activation mechanism (Fig. 7–8) with no apparent influence on PHD activity (Fig. 3). Moreover, we detected complete blocking of insulin-induced HIF-1α accumulation by a global transcription blocker actinomycin D (Fig. 4D). A recent study in human microvascular endothelial cell also reported that PHD activity was not affected in response to insulin [40] supporting our finding of HIF-1α transcription as the prevalent mechanism of insulin-induced HIF-1α accumulation. Cellular ascorbate, iron and oxygen levels regulate PHD activity responsible for hydroxylation of HIF-1α proline residues at 402 and 564. Decrease of any of these should affect PHD activity leading to HIF-1α stabilization. We did not find any significant change in cellular ascorbate level by insulin treatment. By using a hypoxy-probe we also failed to detect any hypoxia like condition within cells (data not shown). Incidentally, depletion of oxygen within the cell by insulin treatment has not been reported so far in the literature. A previous report described increase in cellular iron uptake protein transferrin receptor recycling in 3T3-L1 adipocyte implying increased intracellular iron level by insulin [41]. Since, cellular PHD activity also regulates the other regulatory subunit of HIF isoform HIF-2α, a change in PHD activity should result into accumulation of HIF-2α. However, when we verified HIF-2α level no significant change was detected by insulin treatment (Fig. 1C). All these findings strongly support that insulin promotes HIF-1α accumulation by regulating its transcription. Interestingly, Arg residue at 803position of HIF-1α is hydoxylated by factor inhibiting HIF (FIH) to regulate activity of C-terminal trans-activation domain (CAD) [42]. There is evidence of higher sensitivity of this HIF asparaginyl hydroxylation by low concentration of H_2_O_2_ than prolyl hydroxylation [33]. So far the effect of insulin on FIH has not been reported. It will be interesting to find the effect of insulin on FIH activity.

Mainly, ROS mediated NF-kB activation has so far been reported to regulate HIF-1α transcription [11], [26], [43]. ROS either added as H_2_O_2_ or generated by NOX activator thrombin or by over-expression of subunit NOX4 activates NF-kB to promote HIF-1α transcription [11], [26]. Interestingly, basal expression of HIF-1α is controlled by NF-kB in vivo [27]. In this study we have not found any role of NF-kB in insulin-induced HIF-1α transcription. The deletion of NF-kB binding site did not affect insulin-induced HIF-1α promoter activity (Fig. 5). Subsequently, when ChIP assay was performed using antibody of NF-kB subunit p65 no difference in promoter binding with or without insulin treatment was detected (Fig. 6C). A similar result was obtained by immunofluorescence detection of p65 (Figure S2). In contrast, we identified the critical involvement of Sp1 as a Sp1/Sp3 complex binding to GC-rich region (−68 to −58) of HIF-1α promoter by mutational analysis of promoter (Fig. 5C and 5D), electrophoretic mobility shift assay (Fig. 6A and 6B) and chromatin immunoprecipitation analysis (Fig. 6C). Moreover, mithramycin A inhibited insulin-induced HIF-1α expression (Fig. 6E) by its capacity of blocking Sp1 binding to GC-rich region (Fig. 6D) [37]. So far, it is not clear why ROS generation by different agonists result into different mechanisms for HIF-1α accumulation. However, quantity of ROS formation was shown as one of the critical determinants of adopting specific cellular signaling pathway leading to specific set of gene regulation [44], [45]. Whether, a similar reason is involved in different mechanisms of HIF-1 activation by different cellular sources of ROS should be examined in future study.

Sp1 is a prototypic C-H type zinc finger containing ubiquitously expressed DNA binding protein [36]. It is well known as a ROS-sensitive transcription factor and functions differently in response to various generators of ROS. Arsenic induced ROS-generation was reported to oxidize Sp1 to affect gene expression in promyelocytic leukemia cells [46]. In human alveolar epithelial cell line, H_2_O_2_ promotes Sp1 phosphorylation by inhibiting Ser/Thr protein phosphatase 1 and by activating JNK to reduce Sp1-DNA complex formation [47]. In human hepatic HepG2 cells, insulin increases ROS generation by NOX3 to regulate binding of Sp1 to DNA [48]. Similarly, we also found role of NOX generated ROS in insulin-induced Sp1 activation (Fig. 7–8). In this study we detected ROS as an initiator of signaling cascade activating Sp1 through PI3K and PKC. We found that PKC inhibitor Ro31-8220 blocked Sp1 phosphorylation using antibody generated specifically to detect phosphorylation of threonine residue (453 in human, 452 in mouse and 454 in rat) suggesting involvement of PKC as Ser-Thr kinase in this mechanism [36]. Ro31-8220 also blocked insulin-induced HIF-1α promoter activity (Fig. 7D) and accumulation (Fig. 8E) providing further evidence of the involvement of ROS-initiated PKC activation in insulin-induced HIF-1α transcription. Other study reported several PKC-subtypes are involved in insulin signaling pathway as well as in Sp1 DNA binding activity [49]. It needs further studies to determine which one of these PKC subtypes is involved in this signaling mechanism. A recent report also demonstrated that ROS-generation due to mitochondrial DNA mutations could stimulate HIF-1α transcription via PI3K pathway [50].

A previous study described that IGF-1 regulated HIF-1α expression primarily by posttranslational stabilization mechanism without any effect on HIF-1α mRNA in ARPE cell [23]. Even insulin was reported to control HIF-1α accumulation by a translational mechanism dependent on PI3K/TOR dependent pathway in ARPE cells [21]. In contrast we detected insulin primarily regulates HIF-1α transcription in preadipocyte. A very recent report also demonstrated that insulin could increase HIF-1α mRNA and protein level in 3T3-L1 adipocytes [34] strongly supporting our finding of transcriptional regulation of HIF-1α by insulin. So far it is not clear whether insulin and IGF-1 promote HIF-1α accumulation in retinal epithelial cells by differential mechanisms than other insulin sensitive cell types. Very recently we reported that insulin increased HIF-1α protein and mRNA expression in hepatic cells [22]. When we tested HIF-1α promoter activity in HepG2 cells after insulin treatment a significant increase comparable to increased protein and mRNA level (∼ 2-fold) was detected [22] (Figure S3). Similarly, increased mRNA expression and promoter activity of HIF-1α were detected after insulin treatment in skeletal muscle c2c12 cells (Figure S4). A very recent report also reported increased HIF-1α mRNA level but not HIF-1α protein stability as mechanism of insulin-induced HIF-1α accumulation in human microvascular endothelial cell [40]. All these results strongly suggest that insulin–induced HIF-1α transcription is primarily responsible for HIF-1 activation in different insulin-sensitive cell types including adipocyte.

In this study we revealed a novel mechanism of ROS sensitive transcriptional regulation of HIF-1α but the physiological implication of insulin-induced HIF-1 activation remained unclear. Insulin acts as a growth factor and intimately implicated with cellular energy generation. Increased expressions of glucose transporter (Fig. 2D) and TfR1 [22] as well as TfR1 recycling [41] also suggest the need of the requirement of appropriate amount of nutrients for its critical anabolic activity. However, it needs further study to understand the physiological importance of insulin-induced activation of HIF-1 particularly in adipocyte.

## Materials and Methods

### Reagents

Recombinant insulin (Invitrogen), apocynin, diphenyleneiodonium chloride (DPI) and 2′, 7′-dichlorofluorescin diacetate (DCF-DA) were purchased from Calbiochem. Dimethyloxallyl Glycine (DMOG) was from Cayman Chemical. All cell culture reagents and other reagents were obtained from Sigma, if not mentioned otherwise.

### Cell Culture

Mouse preadipocytic 3T3-L1 cells were routinely cultured in Dulbecco's modified Eagle's medium, supplemented with 10% heat-inactivated fetal bovine serum (Invitrogen), 100 units/ml penicillin, 100 µg/ml streptomycin, and 2 mM L-glutamine. Cells were maintained in a humidified atmosphere containing 5% CO_2_ at 37°C [19].

### Immunoblot Analysis

Nuclear extracts (30 µg) were prepared from 3T3-L1 cells and subjected to SDS-PAGE (7.5%) as described earlier [19]. Proteins were transferred to PVDF membrane and incubated with HIF-1α (1∶2000, Abcam), HIF-2α (1∶2000, Abcam) and actin (1∶1000, SantaCruz) antibodies followed by peroxidase conjugated secondary antibody (1∶5000). Western blot for phospho-Thr-Sp1 (1∶1000, Abcam) and Sp1 (1∶1000, SantaCruz) were performed in cell lysates prepared as described earlier [19]. The specific band was detected by ECL reagent and density of the band was estimated using ImageJ software.

### Detection of Intracellular ROS

Intracellular ROS was detected with 2′, 7′-dichlorofluorescein diacetate (DCF-DA; Calbiochem) as probe [19], [31]. Cells incubated with presence and absence of antioxidants and then incubated with 5 µM DCF-DA in serum-free DMEM for 30 min at 37°C in the dark, then treated with medium alone or insulin as mentioned in respective experiment. After the insulin treatment cells were washed in phoshphate buffer saline (PBS), trypsinized, resuspended in 3 ml PBS, and the intensity of fluorescence was immediately read in fluorescence spectrophotometer at 500 nm for excitation and at 530 nm for emission [19]. Similarly, ROS production was also verified by Fluorescence microscopy using the probe 2′,7′-DCF-DA [19], [31].

### RNA Isolation and Reverse Transcriptase-polymerase Chain Reaction (RT-PCR)

Total RNA was isolated from insulin treated and untreated cells using TriPure reagent (Roche). Semi-quantitative RT-PCR was performed using one tube RT-PCR system (Roche) from 2 µg total RNA. For PCR, following primers were used, HIF-1α: forward - 5′ GGC GGC GAG AAC GAG AAG AAA 3′, reverse - 5′ TCC TCC CCC GGC TTG TTA GG 3′; β-actin: forward - 5′ GAC ATG GAG AAG ATC 3′ and reverse - 5′ GAA TGT AGT TTC ATG 3′. Real-time RT-PCR (Applied Biosystem; 7500 Real Time PCR System) was used to analyze transcripts levels of HIF-1α as described earlier [18]. Briefly, total RNA was isolated using Tripure (Roche, Germany); cDNA was prepared from 5 µg of total RNA using High capacity cDNA Reverse Transcription kit (Applied Biosystems, USA). Real time RT-PCR for HIF-1α was performed using HIF-1α assay mix (Mm01283760_m1 HIF-1α) procured from Applied Biosystems, and results were normalized using actin as an endogenous control [Mouse ACTB(20X) pre developed TaqMan® Assay Reagents]. Program for HIF-1α amplification was 50°C–2 min; 95°C–10 min; increasing cycles of (95°C–15 sec; 60°C–1 min).

### Preparation of Nuclear Extract

Nuclear extracts were prepared from 3T3-L1 cells as described before [5], [19], [31]. Briefly, 1×10^8^ cells were washed with ice-cold phosphate-buffered saline and then with a solution containing 10 mM Tris-HCl, pH 7.8, 1.5 mM MgCl_2_, and 10 mM KCl, supplemented with a protease inhibitor mixture containing 0.5 mM dithiothreitol, 0.4 mM phenylmethylsulfonyl fluoride, and 2 µg/ml each of leupeptin, pepstatin, and aprotinin. After incubation on ice for 10 min, the cells were lysed by 10 strokes with a Dounce homogenizer and the nuclei were pelleted. The pellet was resuspended in a solution containing 420 mM KCl, 20 mM Tris-HCl, pH 7.8, 1.5 mM MgCl_2_, and 20% glycerol, supplemented with the protease mixture described above, and incubated at 4°C with gentle agitation. The nuclear extract was centrifuged at 10,000 *g* for 10 min, and the supernatant was dialyzed twice against a solution of 20 mM Tris-HCl, pH 7.8, 100 mM KCl, 0.2 mM EDTA, and 20% glycerol. Protein concentration was determined using the Bio-Rad reagent with bovine serum albumin as standard.

### Electrophoretic Mobility Shift Assay (EMSA)

EMSA was performed as described before [5], [19], [31] using double stranded radiolabeled probe. Sequences of the sense strands of the oligonucleotide probes used for EMSA were as follows: 5′ GAG AGC AAC GTG GGC TGG GGT GGG 3′ (Sp1-Sense) and 5′ GAG AGC AAC GTG **AA**C TG**A A**GT GGG 3′ (mut Sp1-Sense). To measure DNA-protein interaction, 1×10^5^ cpm of oligonucleotide probe was incubated with nuclear extract (5 µg) and sonicated, denatured salmon sperm DNA (0.5 µg) for 20 min at 4°C in a total volume of 20 µl. The reaction mixture was subjected to electrophoresis (200 V in 0.3× Tris-buffered EDTA solution at 4°C) using 5% nondenaturing polyacrylamide gels. Dried gels were subjected to autoradiography up to 24 h.

### Prolyl Hydroxylase Assay

Prolyl hydroxylase activity was determined by monitoring depletion of 2-oxoglutarate by its post-incubation derivatization with o-phenylenediamine (OPD) to form a product amenable to fluorescence analysis [18], [31]. Briefly, 1 mM DTT, 0.6 mg/ml catalase, 2 OG (500 µM), 200 µM of Peptide (ODD 19 mer peptide HIF-1α 556-574, DLDLEMLAPYIPMDDDFQL, Sigma) and 50 mM Hepes pH 7.5 were mixed at 37°C for 5 min. Concurrently, cytosolic extract (50 µg) and iron were mixed at room temperature for 3 min. The reaction was initiated by addition of cytosolic extract/iron mix to the substrate/cofactor mix (final volume 100 µl). After 5 min, 200 µl of 0.5 M HCl was added to stop the reaction. Derivatization was achieved by addition of 100 µl of 10 mg/ml OPD in 0.5 M HCl and heating at 95°C (10 min). After centrifugation (5 min) supernatant (50 µl) was made basic by addition of 30 µl of 1.25 M NaOH and fluorescence was measured using excitation filter at 340 nm and emission filter at 420 nm.

### Murine HIF-1α Promoter Constructs Preparation

HIF-1α promoter region (−1034 to +339 of transcription start site) was cloned by PCR from mouse genomic DNA using primers containing Kpn1site underlined in forward primer (5′ ATA CAT GGT ACC CAC GAA GTG TTC CTT TG 3′) and Xho1site underlined in reverse primer (5′ ATA CAT CTC GAG AAA GAG ACA AGT CCA 3′). PCR fragment was cloned upstream of luciferase gene in pGL3 basic vector (Promega). Progressive deletions from 5′end were performed by PCR using different forward primers containing Kpn1 site using above-mentioned 1373 nucleotide fragment as template and reverse primer, in pGL3 basic vector. Following forward primers were used for constructing deletion mutants as for −250, 5′ ATA CAT GGT ACC AAG CCA GAC GCA 3′; for −100, 5′ATA CAT GGT ACC AGC TGA CCT CCT C 3′; and for −50, 5′ATA CAT GGT ACC TGG CCG CCT GCG T 3′. GC-rich putative Sp1 binding site was mutated by megaprimer method [19], [31] using 5′ GAG AGC AAC GTG **AA**C TG**A A**GT GGG 3′ primer and −250 to +339 construct as template. All constructs were confirmed by sequencing.

### Transfection by Electroporation Method

Cells (50–60% confluent) were transiently transfected by electroporation using BTX Harvard Apparatus, ECR 630 model using following condition: Voltage: 500V; Resistance: 25 Ohm; Capacitance: 50 µF. After transfection cells were seeded in serum containing media and kept for 24 h to recover. Cells were then treated as required by the experimental demand.

### Promoter Assay

Luciferase activity in cell lysate was assayed using a kit (Promega). As a control of transfection efficiency cells were also transfected with SV40 promoter containing β-galactosidase construct and assayed using a Promega kit [5], [19], [31]. Results are expressed after normalization with β-galactosidase activity.

### Chromatin Immunoprecipitation (ChIP) Assay

ChIP assay was performed as described earlier [51]. After insulin treatment (4 h) cells were fixed for 10 minutes at room temperature by adding 1% formaldehyde. The reaction was quenched by adding glycine (0.125 M). Cells were washed twice with 1× PBS, scraped in 1 ml PBS, pelleted down, resuspended in buffer (3 mM MgCl_2_, 10 mM NaCl, 10 mM Tris-Cl pH 7.4, 0.1% NP-40 and protease inhibitors), kept in ice for swelling and were homogenized. Pelleted nuclei were resuspended in buffer (1% SDS, 10 mM EDTA and 50 mM Tris-Cl, pH 8.1 and protease inhibitors), sonicated in ice for 10s pulses and centrifuged at 10000×g for 15 min at 4°C. The chromatin solution was diluted 10-fold in buffer (0.01% SDS, 1.1% Triton X-100, 1.2 mM EDTA, 16.7 mM Tris–HCl, pH 8.1, 167 mM NaCl, 10 mM PMSF and protease inhibitor) after preclearing by addition of protein A-Sepharose beads for 1 h at 4°C. Then chromatin solution was incubated with anti-Sp1 antibody (4 µg), anti-p65 antibody (4 µg) for 16 h in rotating condition at 4°C. Protein-DNA complexes were immunoprecipitated by protein A- Sepharose beads. RNase A and NaCl (final 0.3 M) were added and incubated at 67°C for reverse cross-linking. Proteinase K (2 µg) was added and then DNA was recovered by phenol: chloroform extraction, precipitated with ethanol, resuspended in 50 µl H_2_O. Association of Sp1 and NFkB in the HIF-1α promoter was examined by PCR using specific primers (Fwd: 5′ACT CGC TCC AGC AGC GCC 3′; Rev: 5′ GGG TTC CCG GAG AGC CAA TG 3′, positions −273 to −11).

### Statistical Analysis

All experiments have been performed at least three times independently with similar results and representative experiments are shown. Densitometric results are normalized with respect to internal controls and are expressed relative to the results of untreated control. Error bars represent standard deviations. Since, experiments regarding transfection were performed by electroporation resulting into variable transfection efficiency, p value had been considered as <0.07.

## Supporting Information

Figure S1 Effect of DPI on insulin-induced HIF-1α mRNA expression. Serum deprived cells were treated with media, insulin (30 nM), pretreated DPI (5 µM) plus insulin, and DPI (5 µM). After 4 h total RNA was isolated and qPCR was performed. Results are obtained from three independent experiments. Error bars represent standard deviation.
(TIF)

Figure S2 Effect of insulin on nuclear translocation of p65. Cells were treated with insulin (30 nM) for 30 min, then washed, fixed, permealized and indirect immunofluorescence assay was performed using p65 antibody (left column). Cells were also stained with Hoechst dye for nuclear staining (middle column) and both images were merged (right column).
(TIF)

Figure S3 Effect of insulin on HIF-1α promoter activity in HepG2 cells. HepG2 cells were transiently transfected with the 250 bp upsteam segment of the HIF-1α 5′-flanking region containing the putative Sp1 binding GC-rich region ligated upstream of the luciferase gene in pGL3-basic vector along with with SV40 promoter-linked β-galactosidase. Luciferase assay was performed after insulin (30 nM) in cell lysates and normalized with β-galactosidase activity. Results are expressed as SD of three independent experiments performed in triplicate.
(TIF)

Figure S4 A. Effect of insulin treatment on HIF-1α mRNA expression in c2c12 cells. Real time RT-PCR was performed from total RNA isolated from cells after 4 h of insulin (10 nM) treatment using mouse specific HIF-1α and β-actin primers obtained from Applied Biosystems. Results are expressed as SD of three independent experiments. **B: Effect of insulin on HIF-1α promoter activity in c2c12 cells**. Cells were transiently transfected with the 250 bp upsteam segment of the HIF-1α 5′-flanking region containing the putative Sp1 binding GC-rich region ligated upstream of the luciferase gene in pGL3-basic vector along with with SV40 promoter-linked β-galactosidase. Luciferase assay was performed after insulin (10 nM) in cell lysates and normalized with β-galactosidase activity. Results are expressed as SD of three independent experiments performed in triplicate.
(TIF)
