# Supplementary figures and images for: Insulin Regulates Hypoxia-Inducible Factor-1α Transcription by Reactive Oxygen Species Sensitive Activation of Sp1 in 3T3-L1 Preadipocyte
Source: PLoS One. 2013 Apr 23;8(4):e62128. doi: 10.1371/journal.pone.0062128 (PMC3633924; doi:10.1371/journal.pone.0062128)

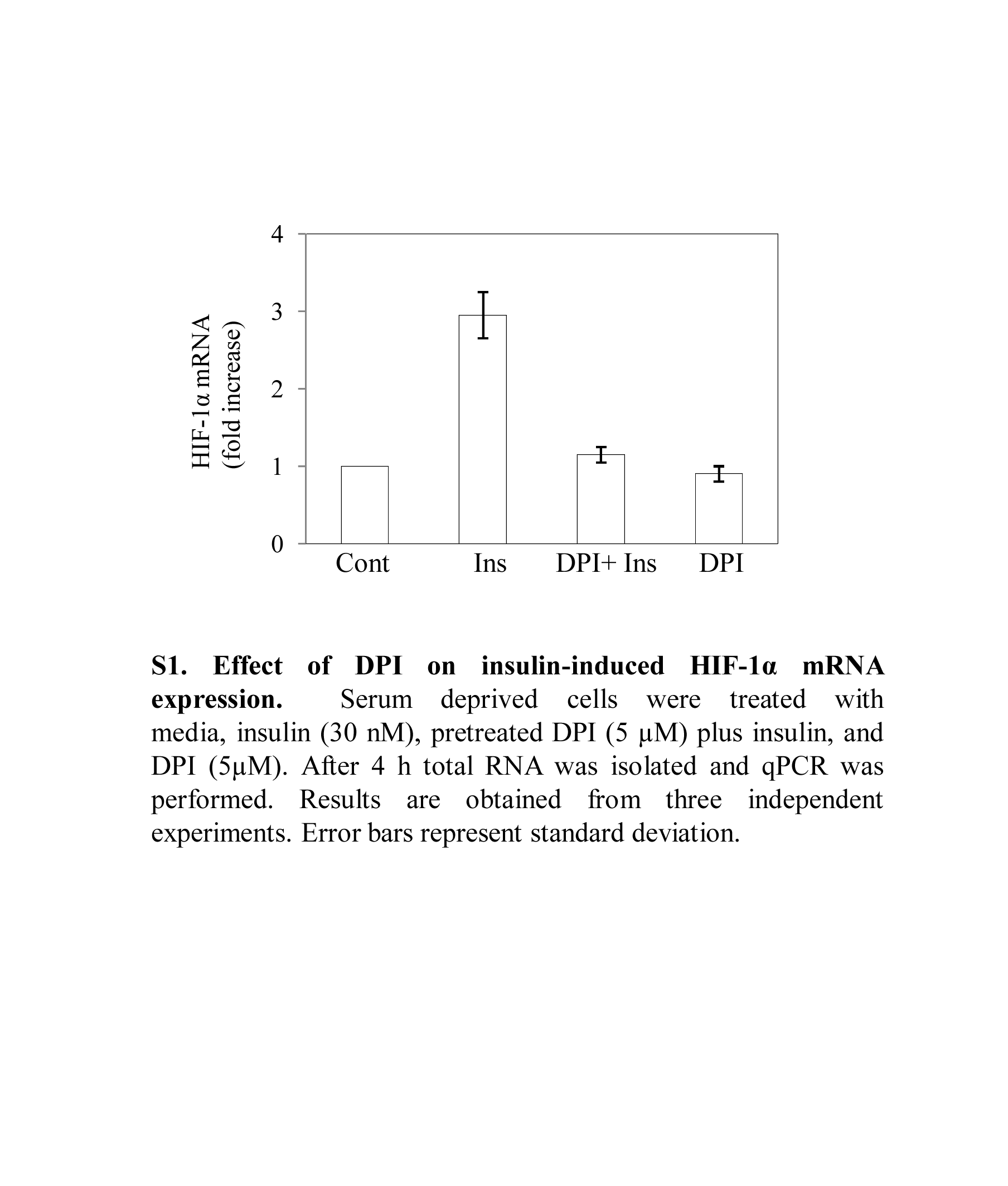

Supplement: Figure S1 — Effect of DPI on insulin-induced HIF-1α mRNA expression. Serum deprived cells were treated with media, insulin (30 nM), pretreated DPI (5 µM) plus insulin, and DPI (5 µM). After 4 h total RNA was isolated and qPCR was performed. Results are obtained from three independent experiments. Error bars represent standard deviation. (TIF) [file pone.0062128.s001.tif]

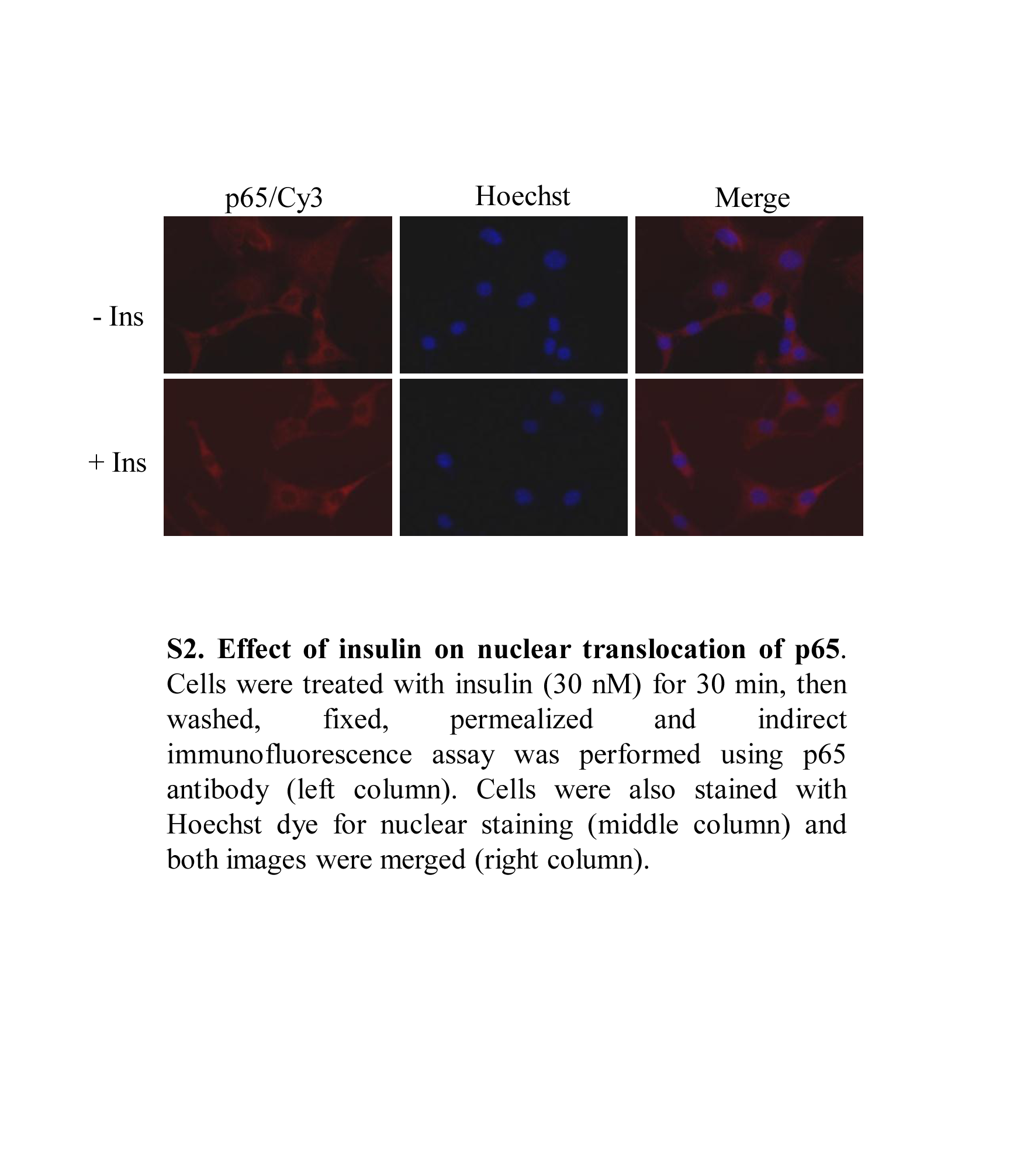

Supplement: Figure S2 — Effect of insulin on nuclear translocation of p65. Cells were treated with insulin (30 nM) for 30 min, then washed, fixed, permealized and indirect immunofluorescence assay was performed using p65 antibody (left column). Cells were also stained with Hoechst dye for nuclear staining (middle column) and both images were merged (right column). (TIF) [file pone.0062128.s002.tif]

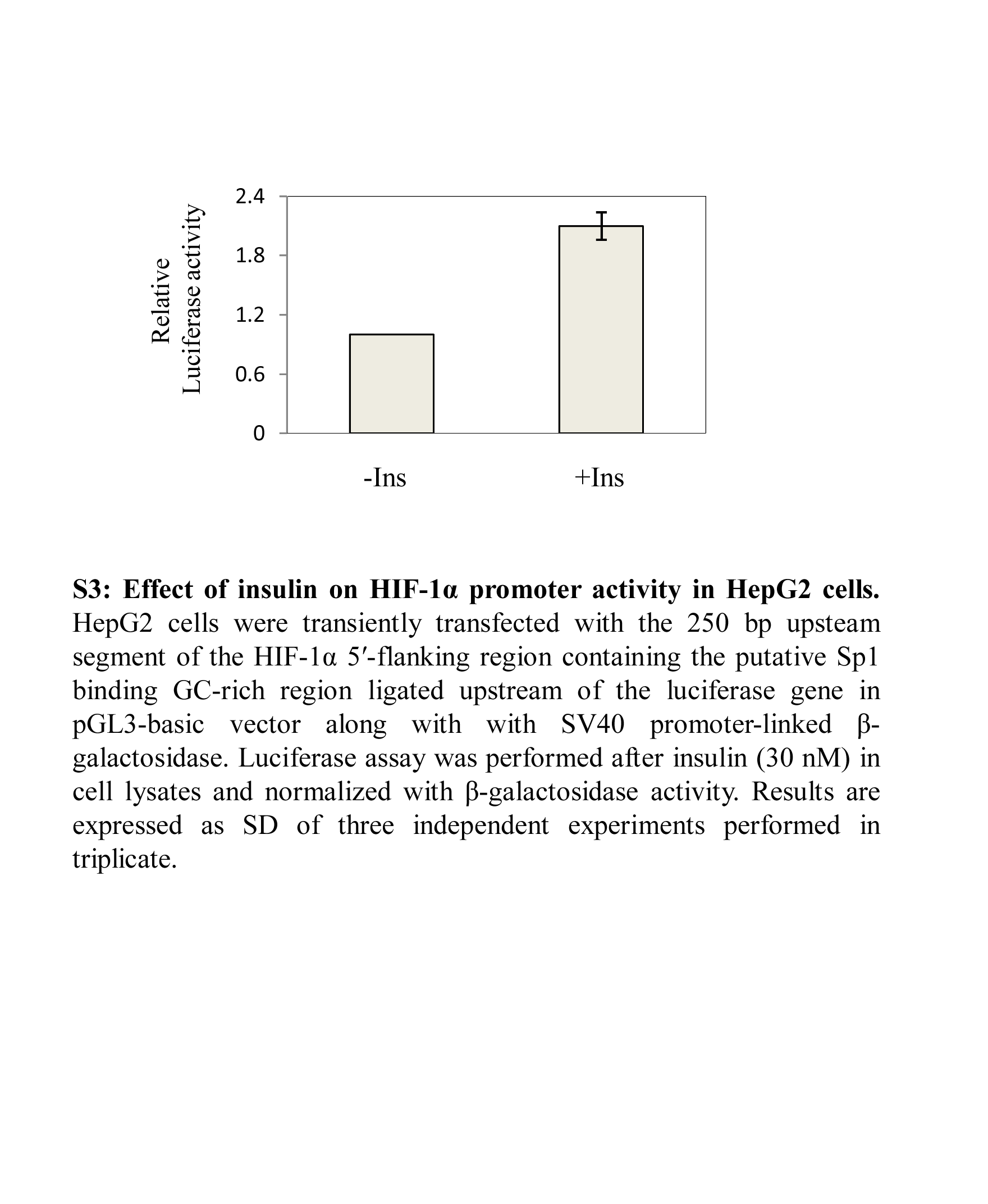

Supplement: Figure S3 — Effect of insulin on HIF-1α promoter activity in HepG2 cells. HepG2 cells were transiently transfected with the 250 bp upsteam segment of the HIF-1α 5′-flanking region containing the putative Sp1 binding GC-rich region ligated upstream of the luciferase gene in pGL3-basic vector along with with SV40 promoter-linked β-galactosidase. Luciferase assay was performed after insulin (30 nM) in cell lysates and normalized with β-galactosidase activity. Results are expressed as SD of three independent experiments performed in triplicate. (TIF) [file pone.0062128.s003.tif]

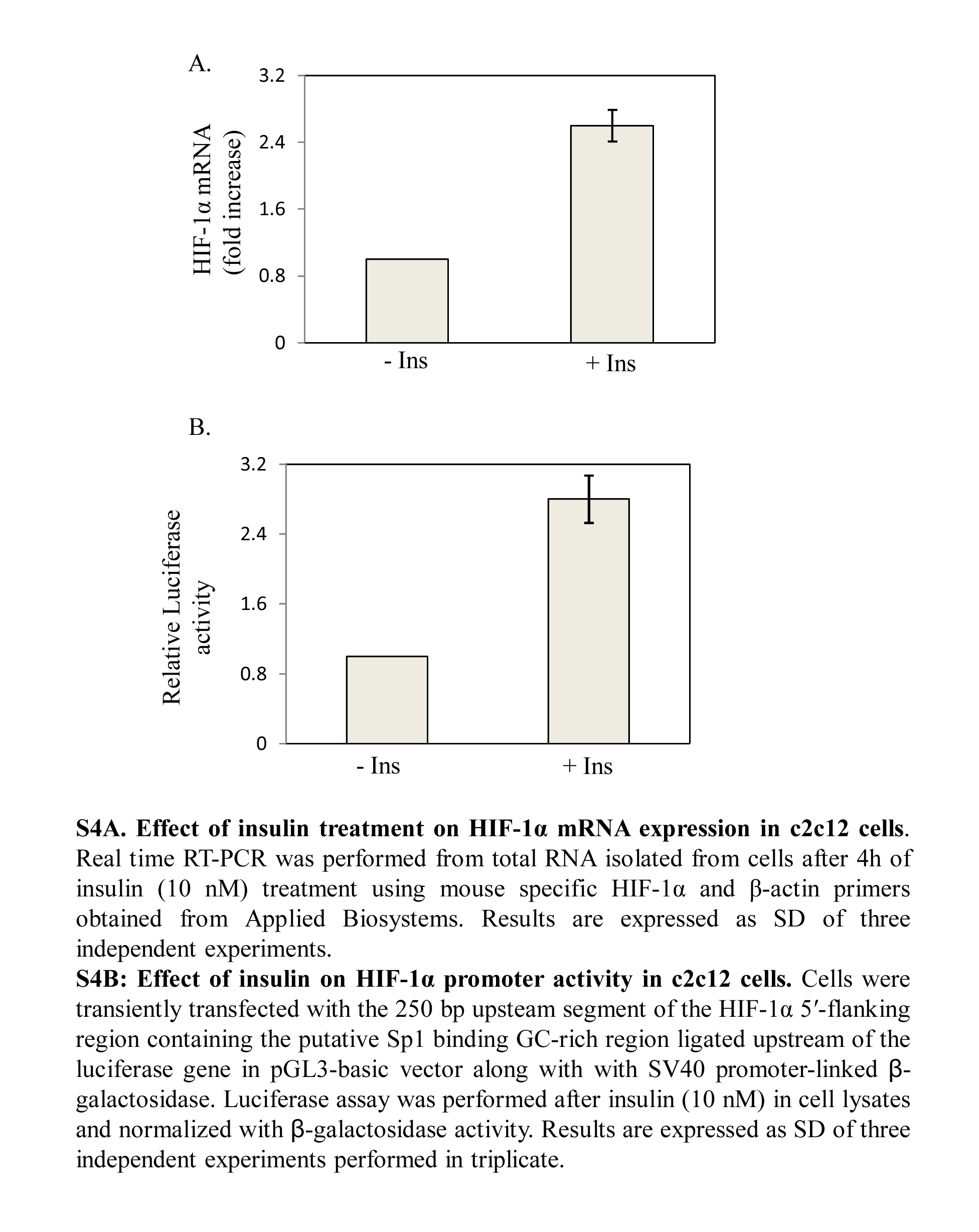

Supplement: Figure S4 — A. Effect of insulin treatment on HIF-1α mRNA expression in c2c12 cells. Real time RT-PCR was performed from total RNA isolated from cells after 4 h of insulin (10 nM) treatment using mouse specific HIF-1α and β-actin primers obtained from Applied Biosystems. Results are expressed as SD of three independent experiments. B: Effect of insulin on HIF-1α promoter activity in c2c12 cells. Cells were transiently transfected with the 250 bp upsteam segment of the HIF-1α 5′-flanking region containing the putative Sp1 binding GC-rich region ligated upstream of the luciferase gene in pGL3-basic vector along with with SV40 promoter-linked β-galactosidase. Luciferase assay was performed after insulin (10 nM) in cell lysates and normalized with β-galactosidase activity. Results are expressed as SD of three independent experiments performed in triplicate. (TIF) [file pone.0062128.s004.tif]
